# Epidemiology of chronic pain in Ukraine: Findings from the World Mental Health Survey

**DOI:** 10.1371/journal.pone.0224084

**Published:** 2019-10-17

**Authors:** Anna Xu, Elizabeth Hilton, Riley Arkema, Nathan L. Tintle, Luralyn M. Helming

**Affiliations:** 1 Cognitive, Linguistics, & Psychological Sciences, Brown University, Providence, RI, United States of America; 2 Department of Psychiatry, Perelman School of Medicine, University of Pennsylvania, Philadelphia, PA, United States of America; 3 Department of Psychological Science, Eastern Connecticut State University, Willimantic, CT, United States of America; 4 Department of Psychology, Dordt University, Sioux Center, IA, United States of America; 5 Department of Mathematics, Computer Science, and Statistics, Dordt University, Sioux Center, IA, United States of America; Iranian Institute for Health Sciences Research, ISLAMIC REPUBLIC OF IRAN

## Abstract

Chronic pain can pose a serious challenge in everyday life for many individuals globally, especially in developing countries, but studies explicitly exploring risk factors of chronic pain beyond demographic characteristics using survey data have been scarce. To address this problem, this study analyzed World Health Organization data on chronic pain in Ukraine to explore demographic, psychological, and treatment perception-related risk factors to chronic pain. We replicated previous reports of older age, female sex, married status, inadequate financial resources, and comorbidity of other physical conditions as significant demographic risk factors for chronic pain diagnosis but not necessarily for severe pain. We also found evidence for psychological risk factors and treatment perceptions as significant predictors for chronic pain diagnosis and its severity. These results provide a first step in examining beyond demographic risk factors for chronic pain diagnosis and severity and, instead, assessing potential psychological risk factors.

## Introduction

The challenges of chronic pain affect many individuals globally, with at least 41% of Europeans in developing countries diagnosed with chronic pain.[1] Despite the prevalence of this illness, two-thirds of patients lack treatment, which in turn impacts the government and societal functions detrimentally.[1,2] While large-scale epidemiological data have provided a wealth of information on demographic risk factors for chronic pain in developing countries, few studies have explicitly explored psychological risk factors of chronic pain and the role of treatment perceptions in its diagnosis and severity using survey-wide data.[3]

Currently, the risk factors for chronic pain diagnosis have consistently included being female, over the age of 40, currently married, with coexisting physical conditions.[2–4] For psychological risk factors, only comorbidity of chronic pain and psychiatric diagnosis and the role of psychological stress on chronic pain have been reported.[3,5–7] However, whether psychiatric diagnosis and non-specific psychological distress are risk factors predictive of chronic pain diagnosis and its severity is less clear. Moreover, how perceptions of medical treatment might impact the trajectory of chronic pain’s severity also remains unclear. While medical treatment stigma and perceived treatment efficacy have been consistently cited as two factors critical to understanding the onset of chronic pain, few studies have used survey data to explicitly examine the link between the perceptions of medical treatment and chronic pain on a larger scale.[8–9]

The purpose of this study was to bridge the gap between theories about chronic pain risk factors that go beyond demographic risk factors and large-scale survey data on chronic pain psychological and treatment perception risk factors for chronic pain diagnosis and its severity using World Health Organization data collected from the Ukrainian population. Ukraine was particularly relevant for this study as many Ukrainians report low satisfaction with the healthcare system and the irregular payment schedule leads to distrust of the medical care system.[10] Moreover, rates of mental health problems in Ukraine are high, and significant somatic symptom complaints have been reported in previous Chernobyl residents, suggesting that somatic symptom complaints may be of particular importance to Ukraine.[11–12]

## Materials and methods

This study was conducted in accordance with the recommendations of the Committees on Research Involving Human Subjects of Stony Brook University as well as the Kiev International Institute of Sociology and the Ukrainian Psychiatric Association internal review boards, with written informed consent from all subjects. All subjects provided written informed consent in accordance with the Declaration of Helsinki. The protocol was approved by the Committees on Research Involving Human Subjects of Stony Brook University as well as the Kiev International Institute of Sociology and the Ukrainian Psychiatric Association internal review boards.

### Sample

Our sample consisted of 1720 randomly selected participants from Ukraine (see Table 1) using a cluster sampling strategy. We used sample weights to adjust for non-response (response rate was 78.3%) as we have done before and is described elsewhere [11]. In short census data was used to ensure the sample was representative of the population on key demographic variables. See [11] for additional details. Trained, professional field staff from the Kiev International Institute of Sociology in collaboration with the Ukranian Psychiatric Association administered face-to-face interviews to participants using the Composite International Diagnostic Instrument 2.0 (CIDI 2.0) as part of the World Mental Health initiative of the World Health Organization. The CIDI 2.0 used composed of twenty-two scales assessing everyday functioning, physical and mental health symptoms, demographic variables (e.g., education, marital status, gender), and social networks, and it is designed to assess DSM-IV disorders.[11] Additionally, prior to conducting the study, to evaluate cultural and conceptual appropriateness of this study and procedure for informed consent, discussion groups with recent immigrants from Ukraine were convened at Stony Brook and a pilot study was conducted in the Kiev metropolitan area. Details of the study design are provided in Bromet et al.[11]

**Table 1:** Sample demographic information with weighted sample.

Weighted sample	
(n = 1720) (%)	
Gender	
Male	45.0%
Female	55.0%
Age (years)	
18–34	30.3%
35–49	28.9%
50–64	20.6%
65+	20.1%
Education	
Primary	9.3%
Secondary	45.8%
Specialized secondary	27.2%
Higher	17.8%
Marital Status	
Never Married	14.7%
Married	59.8%
Previously Married	24.1%
Financial Status	
Adequate SES	32.9%
Inadequate SES	48.6%
Very Inadequate SES	17.9%
Comorbid physical condition	63.40%
Health Stigma	
Low (1–1.99)	33.5%
Moderate (2–2.99)	46.5%
High (3+)	20.0%
Perceived Treatment Efficacy	
None	41.60%
Low	20.70%
Moderate	18.00%
High	19.60%
Psychological Distress (K6)	
Low	71.30%
Moderate	22.50%
High	6.20%

*Note*. Health stigma refers to the degree to which a participant felt embarrassed or ashamed due to present health problems, while Perceived Treatment Efficacy refers to how effective participants felt treatment for a health issue was. Psychological distress refers to the Non-Specific Distress Scale K6 scale which probed non-specific psychological distress (e.g., nervousness, depression). More details on how these variables were defined and scored are noted in the Risk Factors subsection of Assessment of Pain in Materials and Methods.

### Assessment of pain

This study used two assessments of pain (current chronic pain and chronic pain severity) obtained from the Chronic Conditions module in the CIDI 2.0.[13] In particular, a participant was considered experiencing current chronic pain if they responded yes to having at least one of the following: arthritis or rheumatism, chronic back or neck problems, frequent or severe headache, or any other chronic pain within the last 12 months.

All participants indicating chronic pain were then asked questions about the severity of their pain symptoms. If a participant indicated more than one chronic pain condition, the participant was asked to refer to a randomly chosen condition for the questions used to assess their pain symptoms. Chronic pain severity was assessed using a 0 to 10 scale where 0 means no interference and 10 means very severe interference from the condition during the last 12-months in terms of how much the condition or its consequences interfered with each of the following: (1) home management; (2) ability to work; (3) ability to form and maintain close relationships with other people; and (4) social life. The scale provided in the CIDI 2.0 stratified chronic pain into mild (1–3), moderate (4–6), and severe (7–10).

Once participants’ pain experiences were labeled as mild, moderate, or severe, we then separated participants with moderate or severe pain (i.e., those with at least moderate pain severity). In particular, we were interested in assessing risk factors that differentiate participants with severe pain from those with at least moderate pain. In our analyses, we used the subset of participants with moderate or severe pain and created a binary variable that was defined by whether participants had severe pain or not.

### Risk factors

We explored the following demographic risk factors commonly reported for chronic pain: sex, age (18–34, 35–49, 50–64, and +65), education level completed (primary, secondary, specialized secondary, and higher), current marital status (never married, married, and previously married), and financial status (adequate, inadequate, and very inadequate). Financial status, in particular, was derived from a response to a short question about whether or not a family typically (a) did not have enough money for food or clothes (very inadequate), (b) typically have enough money for food, but not clothes (inadequate), or (c) typically had enough money for food and clothes (adequate). In addition to these demographic risk factors, we considered a variety of other mental and physical health factors. Someone was considered to have another comorbid, chronic physical condition (of at least 12 months) if they experienced at least one of the following conditions in the previous 12 months: allergies, stroke, heart attack, heart disease, high blood pressure, asthma, tuberculosis, chronic lung disease, malaria, diabetes, ulcer, thyroid, neurological problem, HIV/AIDS, epilepsy, chronic cold, anemia, kidney disease, liver disease, memory problems, immune system problems, and/or cancer. A 12-month mental health diagnosis included having one of the following: anxiety disorder (social phobia, agoraphobia, generalized anxiety disorder, panic disorder or post-traumatic stress disorder), affective disorder (depression or dysthymia), alcohol use disorder (alcohol abuse or dependence), and intermittent explosive disorder.

We explored health stigma in a manner similar to Alonso et al.[14], which probes how much embarrassment, discrimination, or unfair treatment a participant experienced because of his or her health problems. To assess health stigma, we scored participant responses to two questions in the scale World Health Organization Disablement Assessment Schedule II that was part of CIDI 2.0. These two questions probed the extent to which a participant felt embarrassed about their health problems and how much discrimination or unfair treatment they experienced due to it. Greater scores on this measure indicated that a participant internalized greater stigma towards medical treatment. These scores were then divided into three levels based on the distribution of the sample scores: a score from 1–1.99 indicated low stigma; a moderate score from 2–2.99 indicated moderate stigma; and a score of over 3 indicated high stigma.

We also examined participant’s perceived treatment efficacy (PTE) and divided scores in this measure into four levels. Perceived treatment efficacy indicates the level at which a participant believes current treatment for a medical condition was effective, with greater scores indicating greater belief in the efficacy of the treatment. We scored PTE using the Perceived Treatment Efficacy scale from CIDI 2.0, which consists of two questions probing the extent to which a participant felt seeking a professional was helpful. This scale ranges from 0 to 100 (0 meaning the participant did not perceive treatment to be efficacious). After examining the distribution of PTE scores, the following four levels were created to classify PTE scores: a score of 0 (indicating no PTE); a score from 1–20 (indicating low PTE); a score from 25–49 (indicating moderate PTE); and a score from 50–100 (indicating high PTE). Non-specific psychological distress was measured as a three-level variable (low, medium and high) using the Non-Specific Distress Scale created by Kessler et al.[15]

Finally, we assessed the number of body sites where participants reported experiencing chronic pain by summing the number of locations they indicated experiencing pain. The following locations were considered: neck or back, stomach or abdomen, joints (e.g., arms, hands, legs, or feet), face or jaw or joint below the ear, chest, headaches, and other types of chronic pain.

### Data analysis

We accounted for our clustered sample design and adjusted weighting for non-response rate with the survey package in the statistical analysis software R.[16] For full analytic details of weights and clustering, see Bromet et al.[11] First, we used logistic regression models to calculate odds ratios and 95% confidence intervals to identify risk factors of chronic pain in both unadjusted and adjusted models. The binary outcome measure in these models were either yes (a chronic pain diagnosis is present) or no (a chronic pain diagnosis is not present). Adjusted models included all variables with significant unadjusted associations. Next, we assessed factors associated with severe chronic pain within individuals reporting any moderate to severe chronic pain. For this analysis, we used the binary outcome measure yes (severe chronic pain is present) or no (severe chronic pain is not present). We took a similar approach using both unadjusted and adjusted models, but we additionally used a third adjusted model that included the number of locations of chronic pain. All analyses used a significance level of 0.05 and two-sided tests.

## Results

### Sample characteristics

The sample’s (*n* = 1720) most common demographics were married females with secondary education who had inadequate or very inadequate socioeconomic status. Ages of the participants were generally equally distributed with ages 18–34 having the highest representation of 30.3%. Many individuals with chronic pain reported having other physical conditions (e.g., asthma) (63.4%), no perceived treatment efficacy (41.6%), and low psychological distress (71.3%; Table 1).

### Chronic pain prevalence

The overall prevalence of chronic pain in the Ukrainian sample was 60.4% (1039/1720; Table 2). Within those diagnosed with chronic pain, pain from the neck or back was the most prevalent (40.3%), with most people reporting pain in more than one location (Table 2).

**Table 2:** Survey-weighted proportion of people diagnosed with chronic pain, location of chronic pain and number of locations with pain (i.e. pain types).

Diagnosed with pain?	Frequency	
Yes	60.4%	(1039/1720)
No	39.6%%	(681/1720)
Pain Type	Frequency	
Neck or Back	40.3%	(419/1039)
Joints, Limbs, and Digits	36.6%	(380/1039)
Headaches	29.9%	(311/1039)
Stomach or Abdomen	13.5%	(140/1039)
Chest	10.8%	(112/1039)
Face/Jaw/Joint Below Ear	3.8%	(40/1039)
Other	12.0%	(125/1039)
Number of Pain Types	Frequency	
None	36.8%	(382/1039)
1	23.6%	(245/1039)
2	16.6%	(172/1039)
3	10.5%	(109/1039)
4+	12.6%	(131/1039)

*Note*. Survey-weighted proportion refers to frequency assessed after survey weights have been applied to account for overrepresentation of females, people over the age of 50, and people living in urban settings (see subsection Sample in Materials and Methods).

### Risk factors for chronic pain

Of the risk factors included, we found that most demographic risk factors were associated with chronic pain. These included being female; being over 50 years of age (50–64, and 65+); not having finished high school (primary level education only); being currently married or previously married; and having inadequate or very inadequate socioeconomic status. Furthermore, having at least one other comorbid chronic physical condition, high stigma towards health, moderate or severe psychological distress and most psychiatric disorders were also associated with a significantly increased risk of chronic pain. One exception was alcohol use disorders, which were associated with a lower risk of chronic pain. In the adjusted model, however, only the demographic variables being female, being over 50 years of age (50–64, and 65+) and being previously married remained significant, while all other physical and mental health variables remained significant, except for alcohol abuse (see Table 3 for full results).

**Table 3:** Demographic, physical, psychological, and social risk factors of chronic pain diagnosis and their associated odds ratios (unadjusted and adjusted) as well as the proportion of people in their factor with chronic pain.

	% (x/n)	OR (95% CI)	aOR (95%)
Demographics			
Gender			
Male	46.3% (358/774)	1	1
Female	71.9% (681/946)	2.98 (2.3, 3.9)	2.16 (1.51, 3.09)
Age (years)			
18–34	44.5% (232/521)	1	1
35–49	51.6% (257/498)	1.3 (0.9, 2.0)	1.09 (0.73, 1.64)
50–64	73.3% (260/354)	3.4 (2.4, 5.0)	2.17 (1.42, 3.34)
65+	83.7% (290/346)	6.4 (4.2, 9.8)	4.12 (2.22, 7.66)
Education			
Primary	80.6% (129/160)	3.7 (1.9, 7.3)	0.90 (0.37, 2.19)
Secondary	60.1% (473/787)	1.4 (0.9, 2.0)	1.43 (0.91, 2.23)
Specialized secondary	52.8% (247/467)	1	1
Higher	62.1% (190/306)	1.5 (1.0, 2.1)	1.25 (0.82, 1.92)
Marital Status			
Never Married	43.9% (117/267)	1	1
Married	58.0% (597/1029)	1.76 (1.2, 2.6)	1.25 (0.79, 1.96)
Previously Married	76.5% (324/424)	4.14 (2.7, 6.3)	1.76 (1.08, 2.86)
Financial Status			
Adequate	45.0% (138/307)	1	1
Inadequate	61.6% (514/835)	2.0 (1.4, 2.8)	1.37 (0.92, 2.05)
Very Inadequate	67.9% (384/565)	2.6 (1.7, 3.9)	1.30 (0.78, 2.18)
Other physical condition			
Yes	75.5% (813/1076)	5.9 (4.3, 8.2)	4.18 (2.88, 6.06)
No	34.3% (213/621)	1	1
Health Stigma			
Low (1–1.99)	77.9% (127/163)	1	1
Moderate (2–2.99)	82.7% (187/226)	1.37 (0.69, 2.74)	1.02 (0.50, 2.08)
High (3+)	93.8% (91/97)	4.61 (2.26, 9.40)	3.05 (1.33, 6.98)
Psychiatric Diagnoses			
Anxiety diagnosis			
Yes	84.9% (100/118)	4.0 (1.9, 8.1)	2.40 (1.07, 5.37)
No	58.6% (939/1602)	1	1
Mood diagnosis			
Yes	83.7% (144/172)	3.74 (2.4, 5.83)	2.22 (1.43, 3.46)
No	57.8% (895/1548)	1	1
Alcohol Abuse			
Yes	47.6% (49/104)	0.58 (0.35, 0.96)	1.25 (0.72, 2.17)
No	61.2% (989/1616)	1	1
Intermittent Explosive Disorder			
Yes	70.2% (35/49)	1.56 (0.88, 2.79)	2.36 (1.33, 4.21)
No	60.1% (1004/1671)	1	1
Any Psychiatric Diagnosis			
Yes	72.0% (257/357)	1.9 (1.3, 2.8)	1.92 (1.38, 2.67)
No	57.3% (782/1363)	1	1
Psychological Distress (K6)			
Low (1–1.99)	51.5% (584/1134)	1	1
Moderate (2–2.99)	73.6% (264/358)	2.65 (1.90, 3.69)	1.86 (1.39, 2.50)
High (3+)	86.9% (86/99)	6.19 (3.09, 12.41)	3.25 (1.66, 6.34)

*Note*. The first column (% (x/n)) refers to the percentage of participants in the specified category with chronic pain diagnosis. Column OR refers to odds ratios for chronic pain diagnosis associated with each variable and its 95% confidence interval while column aOR refers to the odds ratio for the specified variable after adjusting for all significant variables from OR.

### Risk factors for moderate to severe chronic pain

In assessing risk factors for severe pain, we found, in our unadjusted model, that only the following psychological risk factors were significantly associated with risk for severe pain of those with at least moderate pain: having other physical conditions, none or moderate perceived treatment efficacy, high health stigma, having a mood diagnosis, and having high psychological distress. After adjusting for other risk factors (see methods), however, only having other physical conditions, none or moderate perceived treatment efficacy, and having a mood diagnosis remained significant (see Table 4).

**Table 4:** Demographic, physical, psychological, and social risk factors of severe chronic pain severity from people with at least chronic pain and their associated odds ratios (unadjusted and adjusted) as well as the proportion of people in the factor with severe chronic pain.

Severe				
		Model 1	Model 2	Model 3
	% (x/n)	OR (95% CI)	aOR (95% CI)	aOR (95% CI)
Demographics				
Gender				
Male	7.6% (10/133)	1	1	1
Female	11.6% (39/340)	1.6 (0.9, 3.0)	1.60 (0.84, 3.04)	1.51 (0.81, 2.85)
Age (years)				
18–34	6.0% (5/76)	1	1	1
35–49	10.4% (11/106)	1.8 (0.4, 7.6)	1.96 (0.49, 7.81)	1.69 (0.40, 7.02)
50–64	7.4% (9/119)	1.3 (0.4, 4.1)	1.20 (0.37, 3.88)	1.02 (0.30, 3.42)
65+	14.6% (25/172)	2.7 (0.8, 9.1)	2.75 (0.80, 9.42)	2.15 (0.57, 8.05)
Education				
Primary	14.9% (11/73)	1.8 (0.7, 5.0)	1.13 (0.38, 3.36)	1.06 (0.37, 3.07)
Secondary	11.7% (27/229)	1.4 (0.6, 2.9)	1.32 (0.61, 2.82)	1.37 (0.67, 2.81)
Specialized secondary	8.9% (8/88)	1	1	1
Higher	4.9% (4/82)	0.5 (0.2, 1.7)	0.42 (0.14, 1.29)	0.44 (0.15, 1.32)
Marital Status				
Never Married	6.0% (2/40)	1	1	1
Married	13.8% (23/167)	1.6 (0.6, 4.1)	1.46 (0.48, 4.40)	1.43 (0.47, 4.37)
Previously Married	9.1% (24/265)	2.5 (0.7, 8.6)	2.01 (0.55, 7.43)	2.06 (0.57, 7.42)
Financial Status				
Adequate	8.4% (4/46)	1	1	1
Inadequate	6.3% (14/217)	0.7 (0.2, 3.2)	0.49 (0.11, 2.11)	0.46 (0.10, 2.03)
Very Inadequate	15.2% (32/210)	1.9 (0.5, 7.7)	1.37 (0.35, 5.32)	1.21 (0.31, 4.66)
Other physical condition				
Yes	11.4% (47/416)	3.2 (1.2, 8.6)	3.63 (1.35, 9.73)	3.18 (1.14, 8.91)
No	3.8% (2/54)	1	1	1
Perceived Treatment Efficacy				
None (0)	9.2% (10/109)	5.97 (1.56, 22.84)	9.23 (2.30, 37.02)	10.83 (2.69, 43.67)
Low (1–20)	6.5% (2/31)	3.07 (0.37, 25.42)	3.74 (0.40, 34.87)	2.47 (0.19, 32.76)
Moderate (25–49)	7.7% (3/39)	4.89 (1.06, 22.58)	5.59 (1.14, 27.36)	6.14 (1.27, 29.83)
High (50–100)	2.5% (1/40)	1	1	1
Health Stigma				
Low (1–1.99)	6.6% (5/76)	1	1	1
Moderate (2–2.99)	10.9% (14/128)	1.67 (0.83, 3.33)	1.50 (0.73, 3.08)	1.40 (0.75, 2.61)
High (3+)	18.3% (11/60)	3.14 (1.20, 8.22)	2.56 (0.97, 6.73)	1.96 (0.75, 5.17)
Psychiatric Diagnoses				
Anxiety diagnosis				
Yes	10.5% (5/47)	1 (0.42, 2.38)	1.15 (0.45, 3.00)	1.16 (0.45, 2.98)
No	10.5% (44/425)	1	1	1
Mood diagnosis				
Yes	20% (18/91)	2.77 (1.58, 4.88)	2.50 (1.42, 4.39)	2.23 (1.26, 3.96)
No	8.2% (31/381)	1	1	1
Alcohol Abuse				
Yes	11.6% (2/18)	1.13 (0.36, 3.55)	1.42 (0.40, 5.11)	1.67 (0.49, 5.70)
No	10.4% (47/454)	1	1	1
Intermittent Explosive Disorder				
Yes	5.6% (1/17)	0.5 (0.15, 1.67)	0.60 (0.16, 2.25)	0.55 (0.14, 2.09)
No	10.7% (48/455)	1	1	1
Any Psychiatric Diagnosis				
Yes	15.2% (22/143)	1.95 (1.02, 3.72)	2.01 (1.03, 3.90)	1.85 (0.95, 3.61).
No	8.4% (28/330)	1	1	1
Psychological Distress (K6)				
Low (1–1.99)	7.2% (16/223)	1	1	1
Moderate (2–2.99)	12.6% (19/151)	1.82 (0.80, 4.16)	1.90 (0.84, 4.32)	1.72 (0.74, 3.96)
High (3+)	20.0% (11/55)	3.19 (1.16, 8.78)	3.20 (1.16, 8.84)	2.54 (0.82, 7.86)

*Note*. The first column (% (x/n)) refers to the percentage of participants in the specified category with chronic pain diagnosis. Model 1 includes odds ratios for each variable with a chronic pain diagnosis. Model 2 includes adjusted odds ratios with all significant variables from Model 1. Model 3 includes all significant variables from Model 2 and accounts for number of pain type.

We investigated the robustness of a third multivariable model that included a count of pain types for each individual. The number of pain types was significantly associated with both moderate (69.3% 1 pain type vs. 87.1% 4+ pain types; OR = 1.35 for each additional pain type, 95% CI: 1.12, 1.63; *p* = 0.003) and severe (6.3% 1 pain type vs. 15.7% 4+ pain types; OR = 1.34 for each additional pain type, 95% CI: 1.14, 1.58; *p* = 0.001). We also found that this model that included pain type count also significantly predicted moderate and severe pain better than our second model that did not adjust for pain type count (*p* < 0.05).

## Discussion

This study explored the prevalence of risk factors for the diagnosis of chronic pain and its severity in the Ukrainian population using nationwide survey data. Using this approach, we found a high rate of chronic pain in Ukraine (60.4%), with the majority of the chronic pain population reporting pain in more than one location. Furthermore, we both replicated previous epidemiological studies highlighting similar demographic risk factors for chronic pain (e.g., age, sex, education, marriage, and having another physical condition) and uniquely found both psychological risk factors and medical treatment perception risk factors for chronic pain. Finally, we present a method for assessing risk factors for severe chronic pain. Specifically, we found that while demographic risk factors were generally associated with chronic pain diagnosis, other risk factors such as treatment perception and psychological risk factors were associated with chronic pain severity.

In terms of demographic variables, we found that, in our adjusted models, being female, older, and previously married were significant in predicting a diagnosis of chronic pain. These findings are consistent with the literature and numerous studies have focused on biological and psychosocial factors of hyperviligancy, sensitivity during menstruation, and increased odds of comorbid anxiety or depression generally accounting for women’s higher risks of chronic pain. [17] However, while most of these demographic risk factors were significant in predicting the diagnosis of chronic pain, these variables were not significantly associated with severe chronic pain in participants reporting moderate to severe pain, suggesting that demographic risk factors may not be as sensitive to predicting more severe forms of chronic pain in diagnosed patients. Nevertheless, our study was able to replicate the importance of demographic variables as risk factors to chronic pain [2–4], which is especially useful in formulating public health-related policies related to chronic pain and designing targeted interventions for women as studies like Bartley and Fillingim [18] suggest that numerous factors such as hormones and different coping strategies affect a woman’s odds of having chronic pain.

Interestingly, we found that having a comorbid chronic physical condition increased odds of both diagnosis and severity. Specifically, one interpretation of our comorbidity results can be related to allostatic load (i.e., the “wear and tear” of the body associated with stress) which has been mentioned as an increasingly important factor in the biopsychosocial model of pain.[19] Here, our results provide evidence of an accumulating allostatic load on the body associated with comorbid physical conditions that increases the risk of illness due to increased bodily stress response.[19–20] In line with such a model of increasing risk of illness due to accumulating allostatic load, we also provide evidence suggesting that such an increased risk of illness from a comorbid condition is not limited to physical conditions. Specifically, we find evidence for psychological risk factors associated with chronic pain diagnosis and severity.

In line with literature discussing the comorbidity of psychiatric diagnoses and chronic pain and theories linking psychological factors to chronic pain, psychological risk factors significantly predicted chronic pain diagnosis and different levels of chronic pain severity.[3,19,22] In particular, we found that all classes of psychiatric diagnoses (except for alcohol abuse) and moderate to high levels of psychological distress significantly increased the risk of chronic pain. While only mood disorder diagnosis, just having any psychiatric diagnosis, and moderate psychological distress extended to predicting increased odds of at least moderate pain severity, only mood disorder diagnosis predicted increased odds of severe pain in those with at least moderate pain severity. Adding to the literature on risk factors for chronic pain, our severity analyses warrant the need to further investigate psychiatric disorders and psychological risk factors that may predict different grades of chronic pain severity by emphasizing increased odds of severe pain with mood disorders. These findings support the association of chronic pain with psychological factors and further substantiate the allostatic load component of the biopsychosocial model of pain.[3,19–21,22–23] It is also worth noting the finding that alcohol abuse was not significant in predicting chronic pain at all, raising the question as to what may make alcohol abuse special compared to other disorders in predicting chronic pain. Though it is hard to infer a causal mechanism relating psychiatric diagnoses to chronic pain, further research can help elucidate the specificity of psychological disorders in relationship to predictions about pain.

Finally, to test theoretical models of treatment perception on chronic pain from the psychology literature, our survey-data approach found that, contrary to our hypothesis, perceptions of low or moderate efficacy from medical treatment decreased the odds of having at least moderate pain. However, of those with at least moderate pain, we found increased odds of experiencing severe pain in those who perceived low or moderate efficacy from medical treatment. We interpret our results to suggest that a less optimistic perspective of treatment efficacy may be, initially, an adaptive coping strategy in dealing with surprises surrounding treatment outcomes. In cases when treatment may not always be effective, those with lower expectations about treatment outcomes may maintain the effort to continue with treatment due to this attenuation of surprise surrounding treatment outcome.[24] On the other hand, in cases when pain is already at least moderately severe, what separates those who experience moderate pain compared to severe pain may be related to how optimistic someone may be about the outcomes of treatment as numerous studies have suggested that higher expectations of treatment can lead to better prognosis of pain.[25–27]

In analyzing the effects of stigma about medical treatment on chronic pain, we were only able to find evidence for increased odds of a chronic pain diagnosis associated with high health stigma; we did not find an effect of health stigma on chronic pain severity. These preliminary results may suggest that patients with some form of pain may not initially seek treatment, leading to increased onset of pain that results in the form of chronic pain. As found in Boersma and Linton’s (2006) study, an individual’s negative expectations of treatment is strongly interrelated to one’s experience of pain. [28] Longitudinal studies exploring the role stigma may play in medical treatment can provide further insight into how stigma may increase the odds of being diagnosed with chronic pain.

While we were able to provide a first step in exploring somatic symptoms in chronic pain, there are several limitations to our approach in examining risk factors for chronic pain. Notably, the directionality of our measures remains ambiguous due to the cross-sectional nature of our study. Future studies using longitudinal designs could better clarify the time sequence of our measures. Additionally, response bias may inflate the memory for the severity of past chronic pain symptoms–caution should be used in interpreting severity results, though real-time data collection may provide a better method of pain assessment.[29] Nevertheless, our study warrants the need to examine different factors beyond demographic risk factors when analyzing risk factors to chronic pain. We also note that while our definition of chronic pain is a reasonable one based on the questions available in the CIDI, additional studies that ask additional questions (e.g., to differentiate between the new ICD-11 chronic pain subtypes of primary and secondary[30]), are needed in the future.

In conclusion, we have reported a high prevalence rate of chronic pain in Ukraine, as well as several risk factors to chronic pain diagnosis and severity. We were able to replicate demographic risk factors to chronic pain diagnosis highlighted in previous epidemiological studies and utilize survey data to further predict chronic pain severity, the influence of psychological risk factors to chronic pain, and the influence of medical treatment perception. As Ukraine is still developing its medical system, it is an ideal time for future physicians and policy makers to use these identified risk factors for chronic pain and its severity to better provide aid to these populations. Given the prevalence and debilitating nature of chronic pain, future studies should probe further into the demographic populations identified in this study as unique events such as Chernobyl may add additional psychological and treatment perception-related risk factors for chronic pain.[12] Furthermore, future studies should consider using a similar method as described in this study to more comprehensively examine risk factors for both chronic pain diagnosis and its severity level.
